# Rhino-Orbital Mucormycosis After COVID-19 Infection in a Patient With Non-Hodgkin’s Lymphoma

**DOI:** 10.7759/cureus.22485

**Published:** 2022-02-22

**Authors:** Tabiha B Hannan, Shrebash Paul, Lovely Barai, Md. Rafiqul Alam, Fazle Rabbi Chowdhury

**Affiliations:** 1 Internal Medicine, Bangabandhu Sheikh Mujib Medical University, Dhaka, BGD; 2 Microbiology, Bangladesh Institute of Research and Rehabilitation in Diabetes, Endocrine and Metabolic Disorders (BIRDEM) General Hospital, Dhaka, BGD

**Keywords:** bangladesh, sars-cov-2, mucormycosis, invasive fungal infections, post covid-19 mucormycosis

## Abstract

Severe acute respiratory syndrome coronavirus-2 (SARS-CoV-2) may be succeeded by a spectrum of complications, including invasive fungal infections (IFIs). Here, we describe a case of rhino-orbital mucormycosis in a recovered coronavirus disease-19 (COVID-19) patient with underlying non-Hodgkin's lymphoma (NHL). Our patient was normotensive, non-diabetic, presenting with multiple non-healing ulcers on different parts of the body. She received high-dose glucocorticoids and antibiotics during her severe COVID-19 illness. Three weeks following COVID-19 detection, she developed progressive rhino-orbital lesion with profuse pus formation, along with pain and redness of the left eye. Histopathology from the lesion revealed mucormycosis. She was treated with Amphotericin B. Unfortunately, the patient died after the first cycle of chemotherapy for NHL. Due to the high chance of mortality, timely clinical suspicion along with microbiological diagnosis is necessary for the early detection of infection. Strong policymaking should also be implicated to revisit the cost effectiveness of available treatments to reduce case fatality.

## Introduction

Severe acute respiratory syndrome coronavirus-2 (SARS-CoV-2) has caused a rapid spread of illness and case fatality since the first case detection in 2019. Due to immunosuppression related to the disease itself, associated co-morbidities, and consequences of treatment, several opportunistic infections in COVID-19 recovered patients have been reported [1,2]. These include invasive fungal infections (IFIs), particularly mucormycosis. The number of mucormycosis cases in recovered patients has been upsurging in India after the second wave of COVID-19 [2].

Mucormycosis is a rare, emerging fungal infection, caused by a group of fungi belonging to the order Mucorales, subphylum Mucoromycotina. This order contains fungi from seven families, among which Rhizopus is the most common, followed by Mucor and Lichtheimia. Other genera include Rhizomucor, Saksenaea, Cunninghamella, and Apophysomyces [3,4]. These are saprophytes, that can be found in air, soil, and decaying objects in nature. Although infections in immunocompetent individuals have been reported, they usually cause infection in immunocompromised hosts [5,6]. The most common presentations are rhino-orbito-cerebral, pulmonary, cutaneous, and disseminated. Isolated renal mucormycosis has also been reported [4]. Here, we describe a case of rhino-orbital mucormycosis in a patient with non-Hodgkin’s lymphoma (NHL).

## Case presentation

A 51-year-old Bangladeshi woman was admitted in February 2021 with complaints of multiple non-healing ulcers in different parts of the body for the last two years. She also complained of low-grade fever, anorexia, and weight loss for the same duration. She did not have any significant past medical, surgical, or family history. In April 2019, the patient developed multiple nodular swellings over different parts of the body, which later on became ulcerated with discharging pus. The ulcer first appeared over the shin of the right leg, and then gradually developed over the lateral aspect of the left leg, both thighs, lower lateral part of the left breast, and lastly, on the left side of her face over one year. She did not have any history of joint pain, oral ulcer, alopecia, fetal loss, genital ulcer, cough, chest pain, epistaxis, bowel or bladder abnormality. Since 2019, she consulted with several physicians as an outpatient and was also admitted to three hospitals for evaluation of her dermatological condition. However, an accurate diagnosis could not be made due to inadequate extensive diagnostic services available in a developing country like Bangladesh. She had been labelled as a case of Pyoderma gangrenosum on the basis of clinical suspicion and was treated with intravenous methylprednisolone for three days and multiple courses of antibiotics including ceftriaxone, co-amoxiclav, flucloxacillin, cefixime, doxycycline for the first 3-4 months without any significant improvement. In 2020, the patient developed cervical lymphadenopathy and underwent fine-needle aspiration cytology (FNAC) from the anterior cervical lymph node, which suggested chronic granulomatous inflammation with caseation. She was then treated with category-1 anti-tuberculous drug therapy (ATT) for six months. After completing her treatment with ATT, the lymph nodes disappeared but her skin lesions persisted and became more aggressive. Before admission to our hospital, a biopsy with histopathology from ulcers over the left leg and face were done twice, but both the reports were inconclusive.

**Figure 1:**
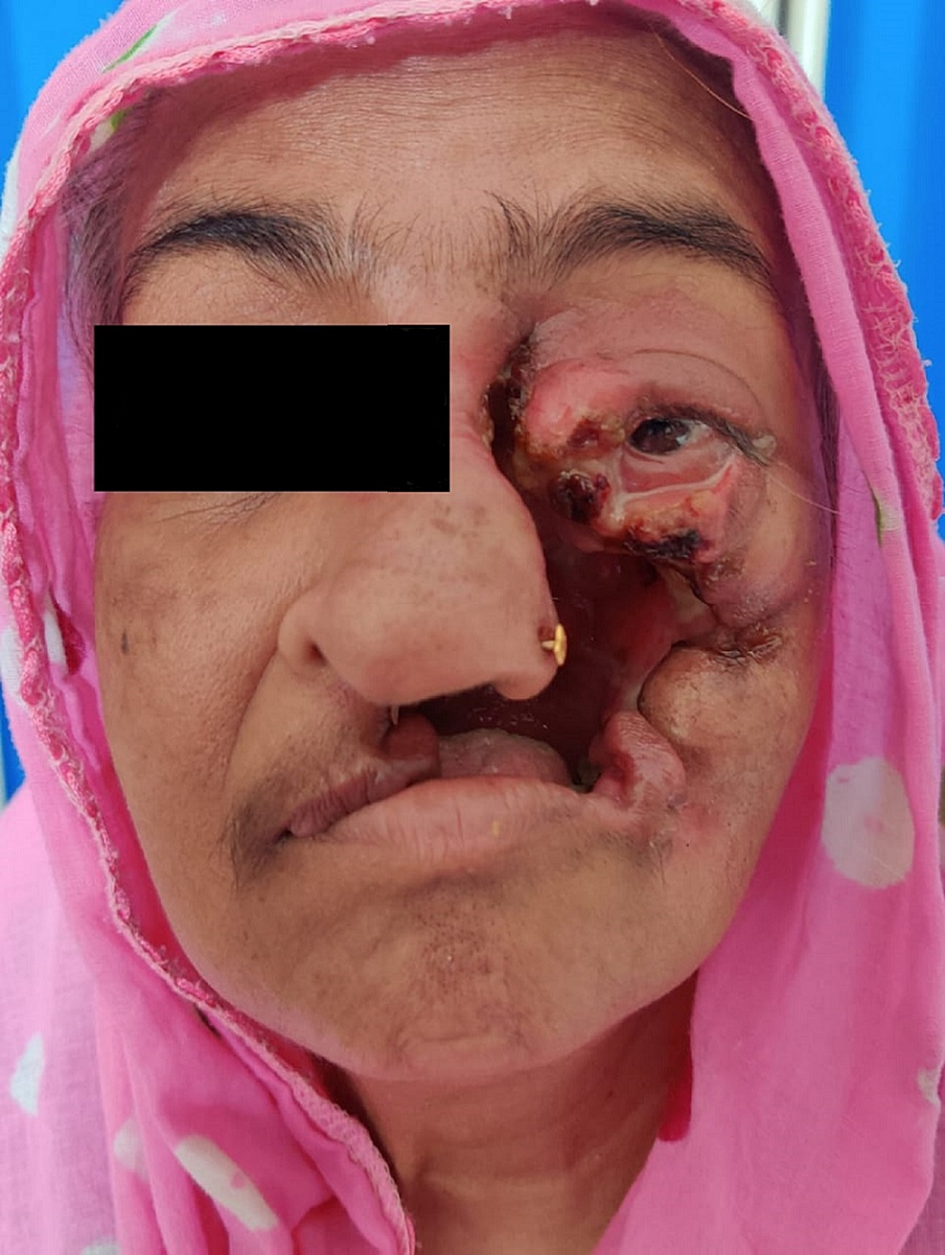
Complete destruction of left maxilla and left upper jaw with loss of teeth and left eye involvement

On admission to our hospital, the patient had moderate pallor and all vital signs were normal. There was no lymphadenopathy or organomegaly. However, she had a grade four ulcer over her left maxillary region measuring about 9×6 cm involving nasolabial fold and nasal wall associated with complete destruction of left maxilla and left upper jaw with loss of teeth (Figure 1). The margin was irregular, the lesion was mildly tender with no visible discharge. Some non-healing grade two ulcers were present involving the right shin, lateral aspect of left leg, both thighs, lower lateral part of the left breast. An extensive grade three ulcer was also present on the lateral aspect of the left leg measuring about 12×6 cm, with an irregular margin, mild tenderness, firm base, and the floor was covered with unhealthy granulation tissue.

**Figure 2:**
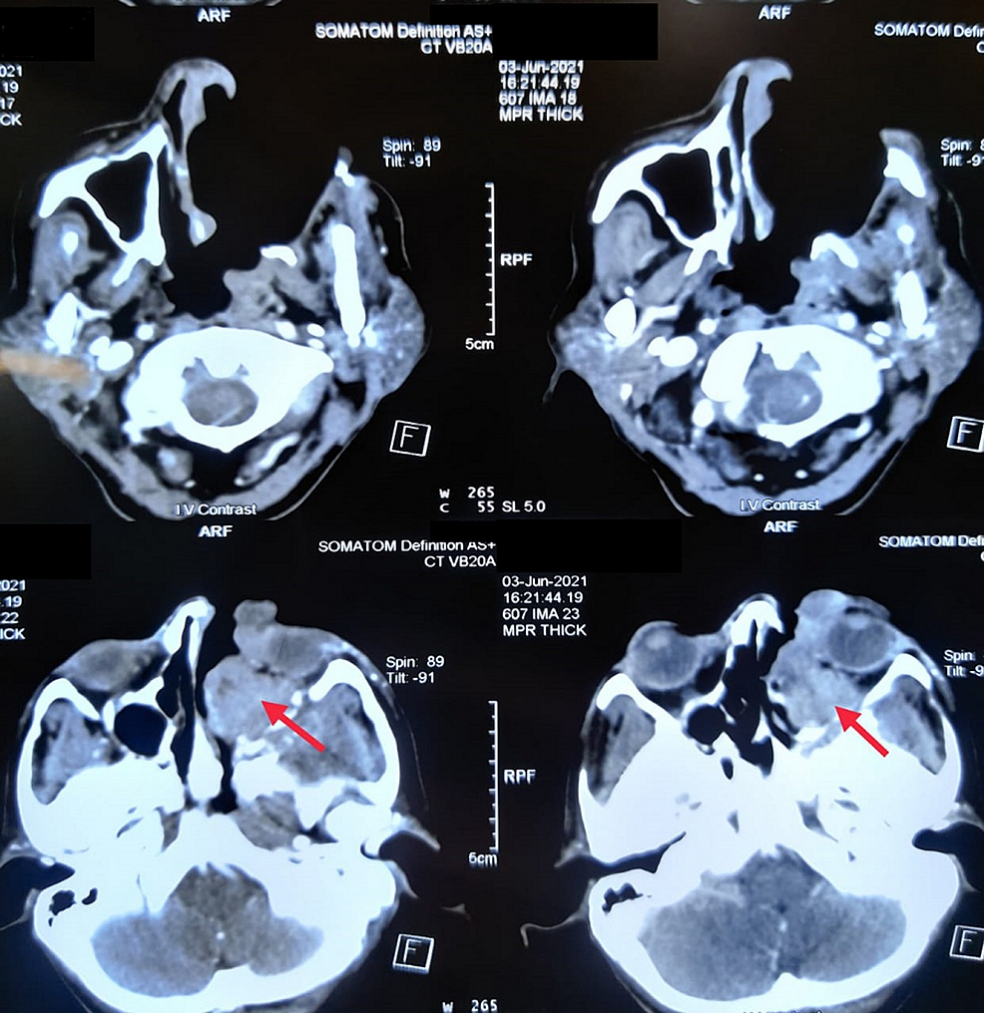
Computed tomography scan of the face showing a left-sided sino-nasal mass surrounding bony destruction and extension to the left orbit

Our working differential diagnoses included deep fungal infection, vasculitis, lepromatous leprosy, and malignancy (cutaneous lymphoma). We underwent extensive investigations as her course of illness was already prolonged due to a diagnostic standstill. Anti-neutrophil cytoplasmic antibodies (ANCAs) were negative, and slit skin smear for leprosy was also negative (Table 1). Biopsies from a skin lesion (left leg) and face were taken, and a histopathology report revealed possible granulomatosis with polyangiitis (GPA). The patient was immediately put on a treatment plan with pulse methylprednisolone followed by oral prednisolone (1mg/kg/day) for six weeks along with intravenous cyclophosphamide (15 mg/kg) at three weeks intervals. She was also transfused with three units of whole blood for correction of anemia. Regular dressing of the ulcers was done, and some improvement was noted in the form of the appearance of red granulation tissue. Unfortunately, after receiving two cycles of cyclophosphamide, she developed fever, cough, and hypoxia (SpO2 88%), and became positive for SARS-CoV-2. Later on, she was transferred to the COVID unit and was treated with 8-10L of high flow oxygen, steroids (dexamethasone 6 mg for ten days), and enoxaparin. Three weeks later, after recovery from COVID-19, she was shifted to the general ward with a more progressive rhino-orbital lesion. She also developed pain and redness of the left eye with conjunctival chemosis and pus formation. A CT scan of the face showed a left-sided sino-nasal mass with surrounding bony destruction and extension to the left orbit involving extra-conal space and the medial rectus, inferior rectus, and inferior oblique muscles (Figure 2). Repeat biopsy was taken from the lesion (face), which concluded diffuse large B cell NHL.

**Table 1:** Baseline and specific laboratory parameters of the patient, including findings of other concomitant infections WBC: White blood cell, RBS: Random blood sugar, SGPT: Serum glutamic pyruvate transaminase, HBsAg: Hepatitis B surface antigen, HCV: Hepatitis C virus, HIV: Human immunodeficiency virus, TPHA: Treponema pallidum hemagglutination, VDRL: Venereal disease research laboratory, ANA: Antinuclear antibody, c-ANCA: Citrullinated anti-neutrophil cytoplasmic antibody, p-ANCA: Perinuclear anti-neutrophil cytoplasmic antibody, AFB: Acid fast bacilli, M/E: Microscopic examination, KOH: Potassium hydroxide

Laboratory tests	Findings	Reference value
Hemoglobin	5.1 gm/dL	13.5 ± 1.3 gm/dL (Female) 15 ± 2 gm/dL (Male)
Total WBC Count	3.35 x 10^9^/L	7.0 ± 3.0 X 10^9^/L
Neutrophil	86%	40-80%
Lymphocyte	05%	20-40%
Monocyte	04%	02-10%
Eosinophil	05%	01-06%
Basophil	00%	<1-2%
Platelet count	2,50,000/mm^3^	150-450 X 10^9^/L
Total circulating Eosinophil	1608/mm^3^	30-350/mm^3^
RBS	4.5 mmol/L	4.1-5.9 mmol/L
SGPT	24 U/L	Male: <50 U/L Female: <35 U/L
Serum Creatinine	1.1 mg/dL	0.5-1.3 mg/dL
Chest X ray P/A View	Normal study	-
Ultrasonography of abdomen	Normal study	-
Microbiological and pathological findings		
HBsAg & Anti HCV	Negative	-
Anti- HIV (1+2)	Negative	-
TPHA & VDRL	Negative	-
ANA	Negative	<1:40
p-ANCA	Negative	0.0-3.5
c-ANCA	Negative	<1:20
Slit skin smear for AFB	Hansen’s Bacilli not found	-
Histopathology from skin lesion	Non-Hodgkin’s Lymphoma	-
M/E of tissue with 20% KOH (Figure 3)	Broad, non-septate, tissue invasive hyphae of Mucormycosis	-
Pus culture from left eye	Methicillin- resistant Staphylococcus aureus and Klebsiella	-

**Figure 3:**
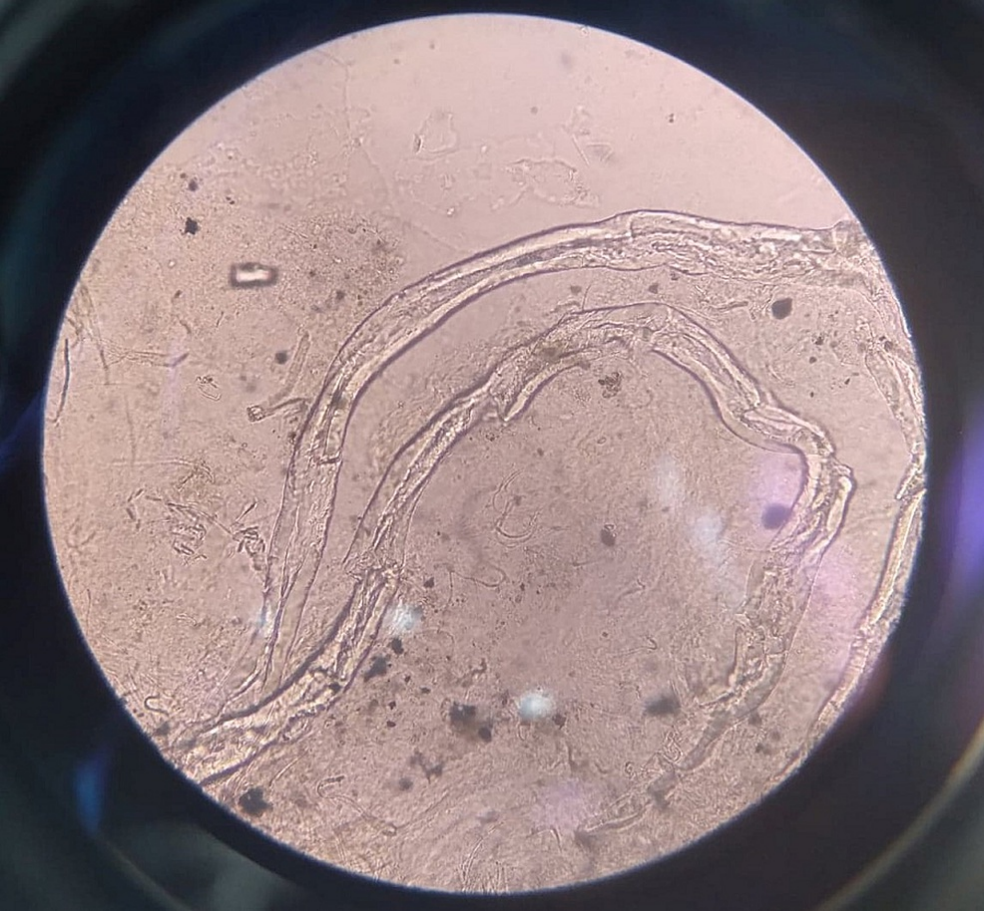
Microscopic examination of the tissue showing broad, irregular, aseptate branching hyphae suggestive of mucormycosis

Immunohistochemistry report confirmed lymphoid granulomatosis. Microscopic examination of the tissue revealed broad, irregular, aseptate branching hyphae suggestive of mucormycosis with no growth in culture (Figure 3). Bacterial culture of pus grew methicillin-resistant Staphylococcus aureus (MRSA) and Klebsiella. The patient was financially unable to afford liposomal amphotericin-B. Therefore, amphotericin B deoxycholate (1 mg/kg-body weight/per day) and injectable meropenem was started along with regular surgical dressing. Chemotherapy was started thereafter, but unfortunately, after receiving the first cycle of chemotherapy, the patient succumbed to death.

## Discussion

Mucormycosis is typically caused by inhalation of fungal sporangiospores from the air, that germinate in the nasal mucosa and paranasal sinuses. Gastrointestinal, cutaneous and disseminated mucormycosis can less commonly be acquired by ingestion and direct inoculation through disrupted skin [4]. In our patient, there were multiple ulcers with bone destruction, which may have been the route of entry for the organism.

Multiple risk factors have been associated with mucormycosis in recovered COVID-19 patients, e.g., diabetes mellitus, HIV infection, hematological malignancy, patients receiving treatment with high dose glucocorticoids, antibiotics, cytotoxics, chemotherapeutic agents, desferrioxamine therapy, organ transplantations, stem cell transplantation, unhygienic oxygen delivery system, etc. [7,8]. COVID-19 itself causes impaired cell-mediated immunity and lymphopenia, which in turn results in decreased levels of CD4+ and CD8+ T cells, that act as an opening gate for opportunistic fungal infections [9]. Although our patient was non-diabetic throughout the course of illness, diabetes mellitus has been reported to be the most common risk factor [10]. Diabetes mellitus can impair the chemotactic activity of neutrophils, causing defective phagocytosis and macrophage activation, which leads to impaired innate immunity. COVID-19 can cause β cell damage causing a hyperglycemic state, which in turn causes acidosis predisposing to mucormycosis. Besides, ferritin levels are increased in COVID-19 as a result of inflammatory response. This unbound free iron aids in the proliferation of fungus. Various immunomodulators have been used in the treatment of severe COVID-19 including tocilizumab, bevacizumab, corticosteroids, which can cause profound immunosuppression and ultimately make way for opportunistic infections including mucormycosis [8,11]. Other factors that may contribute to the development of mucormycosis include virus-induced lymphopenia and endothelitis. Adhesion and penetration of Mucorales may be precipitated by widespread endothelitis [8].

Our patient was diagnosed with NHL and during her COVID-19 illness, she received high dose glucocorticoids too, altogether facilitating the development of aggressive fungal infection. A number of recent but sporadic invasive fungal infections have been reported in recovered COVID-19 patients, including COVID-19 associated pulmonary aspergillosis (CAPA) and COVID-19 associated mucormycosis (CAM) [12]. Mucormycosis in a non-immunocompromised patient has been reported in Bangladesh during the pre-COVID era [6]. Here we report a well-documented case of post-COVID-19 mucormycosis in a patient with underlying NHL.

Management of mucormycosis has been challenging because of multiple site involvement, infection with different species, and delayed diagnosis due to the rarity of the disease. The basic management principles are antifungals, urgent surgical debridement, and correction of risk factors as much as possible [13]. Amphotericin B is the first choice of treatment, because of its highest effect in combating almost all of the species. Posaconazole is an effective alternative [14]. Novel agents, such as isavuconazole have also been implicated in the treatment of mucormycosis. However, voriconazole, fluconazole, and echinocandins have no proven role against fungal spores. Early diagnosis and treatment initiation is crucial to combat the high fatality in these cases. Therefore, policymakers should be more vigilant regarding the prevention of this complication and the availability of the best therapy, amphotericin B should be made possible within the reach of low- and middle-income countries of the world.

## Conclusions

Mucormycosis in post-COVID-19 patients has drawn much attention due to its fulminant course and high mortality. Judicious use of immunomodulators, particularly corticosteroids is pivotal in preventing this complication. Injudicious use of antibiotics may also contribute to this catastrophe. Clinicians must be aware of secondary opportunistic infections in post-COVID-19 patients, especially in patients with immunosuppressed conditions, so that early diagnosis and treatment initiation can be achieved without any delay. Policymakers should also contribute to reforming diagnostic facilities in developing countries so that these invasive infections with high mortality rates do not remain undiagnosed. Also, the cost of the drugs should be revisited so that the best therapy can be started without any hindrance.
